# Genome Sequence of Lacticaseibacillus rhamnosus Strain NCDC610, Isolated from a Traditional Cereal-Based Fermented Milk Product (Raabadi)

**DOI:** 10.1128/MRA.00672-21

**Published:** 2021-11-11

**Authors:** Syed Azmal Ali, Ashish Kumar Singh, Sudhir K. Tomar, Pradip Behare

**Affiliations:** a Proteomics and Cell Biology Laboratory, Animal Biotechnology Center, ICAR, National Dairy Research Institute, Karnal, Haryana, India; b Dairy Technology Division, ICAR, National Dairy Research Institute, Karnal, Haryana, India; c National Collection of Dairy Cultures Laboratory, Dairy Microbiology Division, ICAR, National Dairy Research Institute, Karnal, Haryana, India; University of Arizona

## Abstract

We announce the draft genome sequence of Lacticaseibacillus rhamnosus NCDC610, an isolate from an Indian traditional cereal-based fermented milk product (Raabadi). The genome size of Lacticaseibacillus rhamnosus NCDC610 is 2.91 Mb with the assembled sequence, and the genome consists of 67 contigs.

## ANNOUNCEMENT

Screening of lactobacilli for technological properties provides an opportunity to select potential strains for preparation of functional food (1, 2). Some species of lactobacilli are natural inhabitants of the human gastrointestinal tract and possess the ability to tolerate its harsh environment (3), while others are endowed with industrially important traits suitable for food applications. High-throughput-based microbial genomic and proteomic studies have identified hundreds of genes and proteins in lactobacilli that are associated with metabolic pathways and related physiological functions (4–8). In addition, the ability of lactobacilli to produce phytase enzymes, which degrade the antimicronutrient phytic acid present in cereal-based products, and to convert inorganic zinc to an organic form makes them suitable candidates for developing micronutrient-fortified foods (9–14).

Our isolate of Lacticaseibacillus rhamnosus NCDC610 (previously designated RSI3) has been found to exhibit probiotic properties such as acid and bile tolerance, bile salt deconjugation, cell surface hydrophobicity, antimicrobial activity, and β-galactosidase activity (10). This strain also showed phytase activity and was found to be useful for developing a pearl millet-based fermented dairy food (11).

The isolate was selected using the single colony picking method (10, 11). The culture was grown overnight at 37°C in MRS broth (Becton, Dickinson and Company, Wokingham, Berkshire, England). Genomic DNA was extracted with the UltraClean microbial DNA isolation kit (Mo-Bio Laboratories, Cambridge, England) and purified with the Isolate II PCR and gel kit (BIO-52060; Bioline, Dublin, Ireland) according to the manufacturer’s instructions. Genomic DNA libraries were prepared using a Nextera XT library preparation kit (Illumina, San Diego, CA, USA) following the manufacturer’s protocol with the following modifications: 2 ng of DNA instead of 1 ng was used as the input, and the PCR elongation time was increased from 30 s to 1 min. Libraries were sequenced on the Illumina HiSeq 2500 platform using a 250-bp paired-end read protocol. Read quality was assessed using FastQC v0.11.7 (15). *De novo* assembly was performed with KmerGenie v1.6982 (16), Velvet v1.2.10 (17), SSPACE v3.0 (18), and GapFiller v1-10 (19). Annotation was performed using the NCBI Prokaryotic Genome Annotation Pipeline (PGAP) v4.3 (20). The final draft genomes were estimated to be ≥95% complete, with ≤3% contamination, using CheckM v1.0.12 (21). Default settings were used for all software.

The present study identified a total of 3,012,227 high-quality reads, with a GC content of 46%. The final assembly of strain NCDC610 consisted of 67 contigs, with an *N*_50_ value of 129,063 bp, a maximum contig size of 311,268 bp, and a genome size of 2.91 Mb. The total numbers of coding genes and protein-coding regions in Lacticaseibacillus rhamnosus NCDC610 are 2,825 and 2,712, respectively. We identified 53 RNAs, including 2 rRNAs in the coding gene regions (one 16S rRNA and one 23S rRNA), 48 tRNAs, and 3 noncoding RNAs. Furthermore, 60 pseudogenes and 60 non-protein-coding sequences were determined. The genome was mapped with 30× coverage and also contained one copy of CRISPR arrays (13).

The draft whole-genome result suggests that the identified genes are involved in metabolic processes, genetic and environmental information processing, and cellular processes, which assist the NCDC610 strain in exhibiting probiotic features and acting as starter cultures or adjunct cultures for the manufacture of fermented food products and beverages.

### Data availability.

The whole-genome shotgun project for Lacticaseibacillus rhamnosus strain NCDC610 (RSI3) has been deposited in DDBJ/ENA/GenBank under the accession number JADDUJ010000000 and the SRA accession numbers SRR12887909 and SRR16301660.
